# Isolated thymic metastasis of breast cancer 10.5 years after surgery: a case report

**DOI:** 10.1186/s44215-025-00236-z

**Published:** 2025-11-29

**Authors:** Makoto Tomoyasu, Wataru Shigeeda, Yuka Kaneko, Ryuichi Yoshimura, Hironaga Kanno, Ryotaro Endo, Hiroyuki Deguchi, Hajime Saito

**Affiliations:** Department of Thoracic Surgery, School of Medicine, Iwate Medical University, 2-1-1 Idai-dori, Yahaba, 028-3695 Shiwa, Iwate Japan

**Keywords:** Thymic metastasis, Breast cancer, Asynchronous, Isolated

## Abstract

**Background:**

As breast cancer grows slowly, recurrences more than 10 years after surgery are not uncommon. However, isolated metastasis to the thymus without extra-mediastinal lesions is extremely rare.

**Case presentation:**

A 51-year-old woman underwent right mastectomy for right breast cancer 10.5 years ago. Following surgery, she received adjuvant chemotherapy, radiotherapy, and 10 years of anti-estrogen therapy, with no evidence of recurrence during that period. However, due to a slow upward trend in carcinoembryonic antigen levels, PET imaging was performed and revealed an irregular tumor measuring 20 × 27 × 75 mm in diameter. This consisted of both cystic and solid components, and was located within the thymus, just above the left brachiocephalic vein, with an abnormal 18 F-fluorodeoxyglucose uptake. The tumor was confined to the thymus, with no lesions were detected in the lungs or any other organs. Differential diagnoses included thymic hyperplasia, thymoma, thymic carcinoma, and malignant lymphoma. We performed thymectomy via median sternotomy for both diagnosis and treatment. Immunohistochemical staining confirmed isolated thymic metastasis of breast cancer. The patient is currently being treated postoperatively with aromatase inhibitor and cyclin-dependent kinase 4/6 inhibitor.

**Conclusion:**

We present a surgical case of an extremely rare asynchronous, isolated thymic metastasis that occurred over 10 years after initial breast cancer surgery. This case underscores the importance of considering thymic metastasis in the differential diagnosis of anterior mediastinal tumors in patients with a remote history of breast cancer.

## Background

It is extremely rare for breast cancer to metastasize to the thymus. In a 2019 report, Di Micco et al. summarized rare metastasis sites associated with breast cancer, and found only three cases of metastasis from breast cancer to the thymus in the literature [1–4]. A subsequent literature review revealed that, even when the 2023 study by Aleem et al. [5] was included, only four cases could be identified. Despite incorporating the reports by Sakaguchi et al. in 2006 on metastasis to the thymic lymph nodes [6], Moretto et al. in 2013 on metastasis from breast cancer to thymic epithelial tumors [7], and the two cases reported by Yamashita et al. in 2020, which were presumed to be metastasis to the thymus [8], the total number of cases remains at only eight. It is also noteworthy that merely two among these cases exhibited isolated metastasis to the thymus after a protracted period subsequent to a surgical intervention for breast cancer [4, 8]. In this report, we present the findings for a surgical resection case of isolated thymic metastasis that occurred 10.5 years after the initial breast cancer surgical intervention.

## Case presentation

**Fig. 1:**
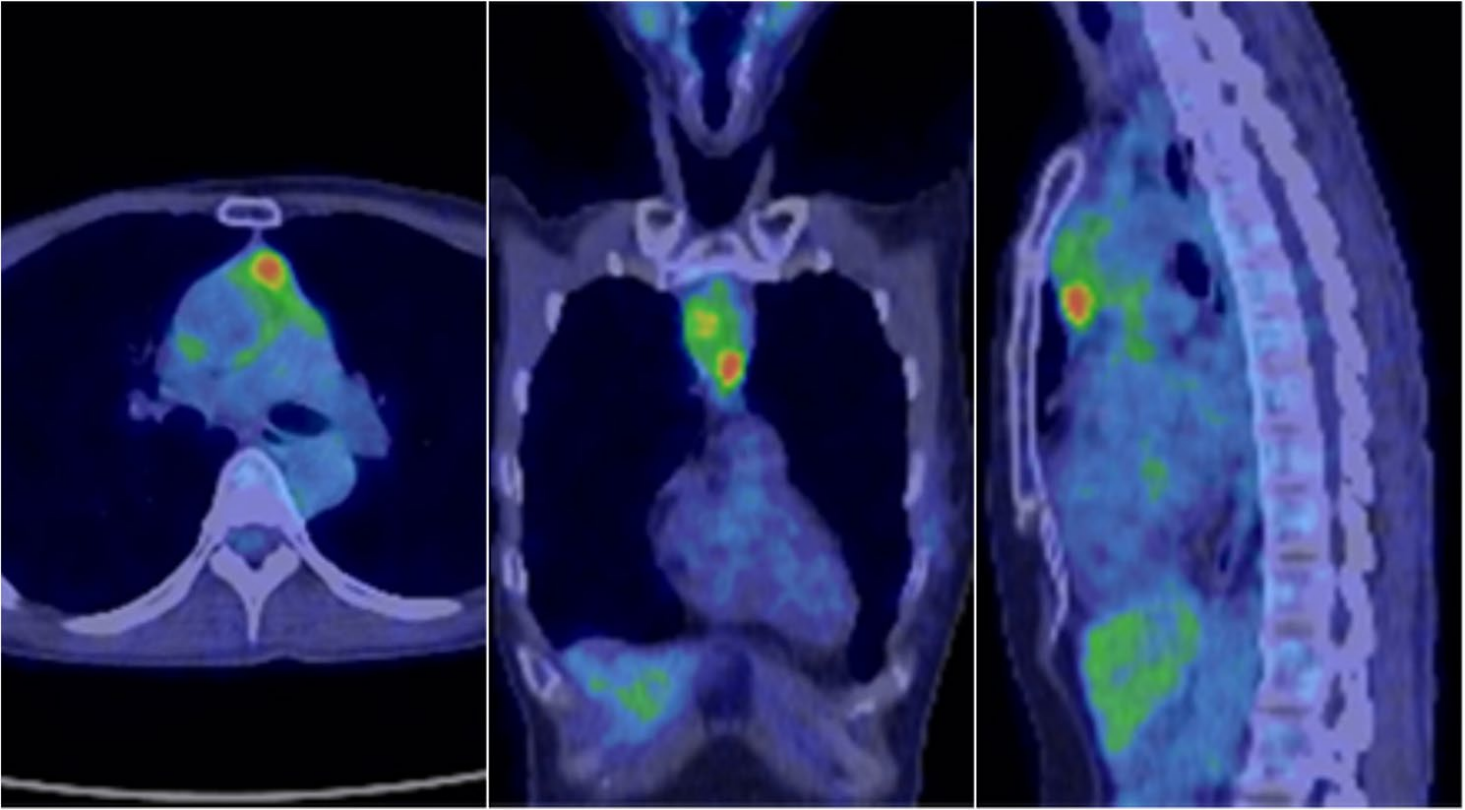
FDG-PET showed substantial accumulation of FDG in the solid components of the tumor (SUVmax = 4.54)

The patient was a 51-year-old female who underwent a right mastectomy and level II axillary lymph node dissection for right breast cancer 10.5 years ago. The pathological diagnosis revealed invasive micropapillary carcinoma measuring 95 × 16 mm (invasion size: 42 × 15 mm) with positive fat invasion, lymphatic invasion 3+, venous invasion 2+, and positive ductal spread. The TNM classification was pT2aN2aM0, Stage IIIA. In the context of immunohistochemical staining, the estrogen receptor (ER) was found to be positive, with an Allred score of PS5 + IS3 = 8. The progesterone receptor (PGR) also demonstrated a positive reaction, with an Allred score of PS2 + IS2 = 4. The human epidermal growth factor receptor 2 (HER2) score was determined to be 1+, while the Ki-67 index was 30%. The patient underwent three courses of FEC (fluorouracil + epirubicin + cyclophosphamide) therapy as adjuvant chemotherapy, which was followed by five courses of TAC (docetaxel + doxorubicin + cyclophosphamide) therapy. Furthermore, postoperative adjuvant radiotherapy with a total dose of 50 Gy in 25 fractions was administered to the right chest wall and supraclavicular lymph node region with the aim of reducing local and regional recurrence. Thereafter, she underwent a 10-year course of oral tamoxifen (TAM) therapy. Computed tomography (CT) performed five years after the initial surgical intervention revealed no evidence of local recurrence or metastasis to any other organs. Additionally, no tumor lesions were identified in the anterior mediastinum. Over the subsequent 10-year period, no recurrence was observed. However, 10.5 years after surgery, carcinoembryonic antigen (CEA) levels gradually increased over time to 4.0 ng/mL (reference level: ≤3.4 ng/mL). As a result, she was further evaluated by 18 F-fluorodeoxyglucose positron emission tomography (FDG-PET), which revealed multiple abnormal FDG accumulations in the thymus, and a maximum standardized uptake value (SUVmax) of 4.54 (Fig. 1). With the exception of the thymus, no abnormal accumulation of FDG was observed in the FDG-PET.

**Fig. 2:**
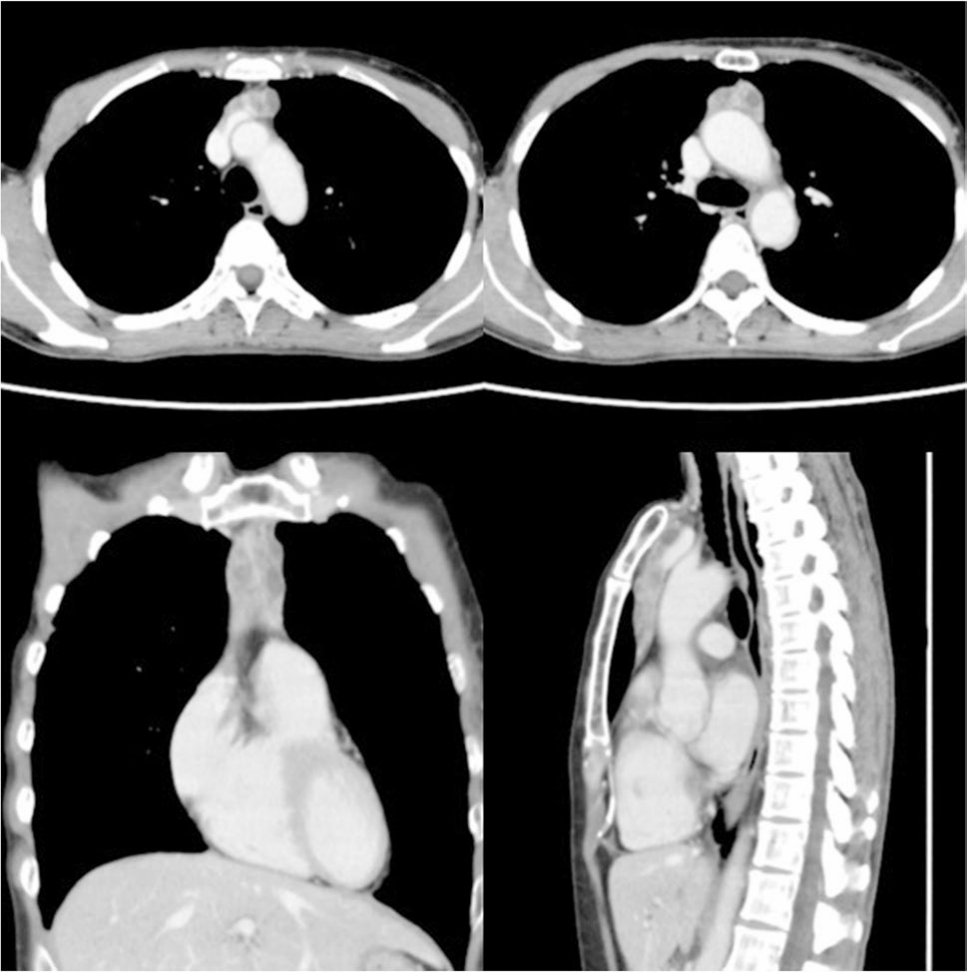
CT scan revealed multiple nodules containing a mixture of solid and cystic components in the thymus, indicating possible invasion into the left brachiocephalic vein

Contrast-enhanced CT revealed a spindle-shaped tumor measuring 20 × 27 × 75 mm that had progressed in a manner that resulted in replacement of the thymus. The lesion consisted of a solid component with heterogeneous contrast enhancement and multiple cystic areas extending from just above the left brachiocephalic vein to the level of the ascending aorta and main pulmonary trunk (Fig. 2). With the exception of a slight increase in CEA, all tumor markers, including the cancer antigen 15 − 3 (CA15-3), squamous cell carcinoma antigen (SCC-Ag), cytokeratin 19 fragment (CYFRA 21 − 1), neuron-specific enolase (NSE), pro-gastrin-releasing peptide (ProGRP), alpha-fetoprotein (AFP), and soluble interleukin-2 receptor (sIL-2R), were within normal ranges. The differential diagnosis under consideration included thymoma, thymic carcinoma, thymic hyperplasia, and malignant lymphoma.

**Fig. 3:**
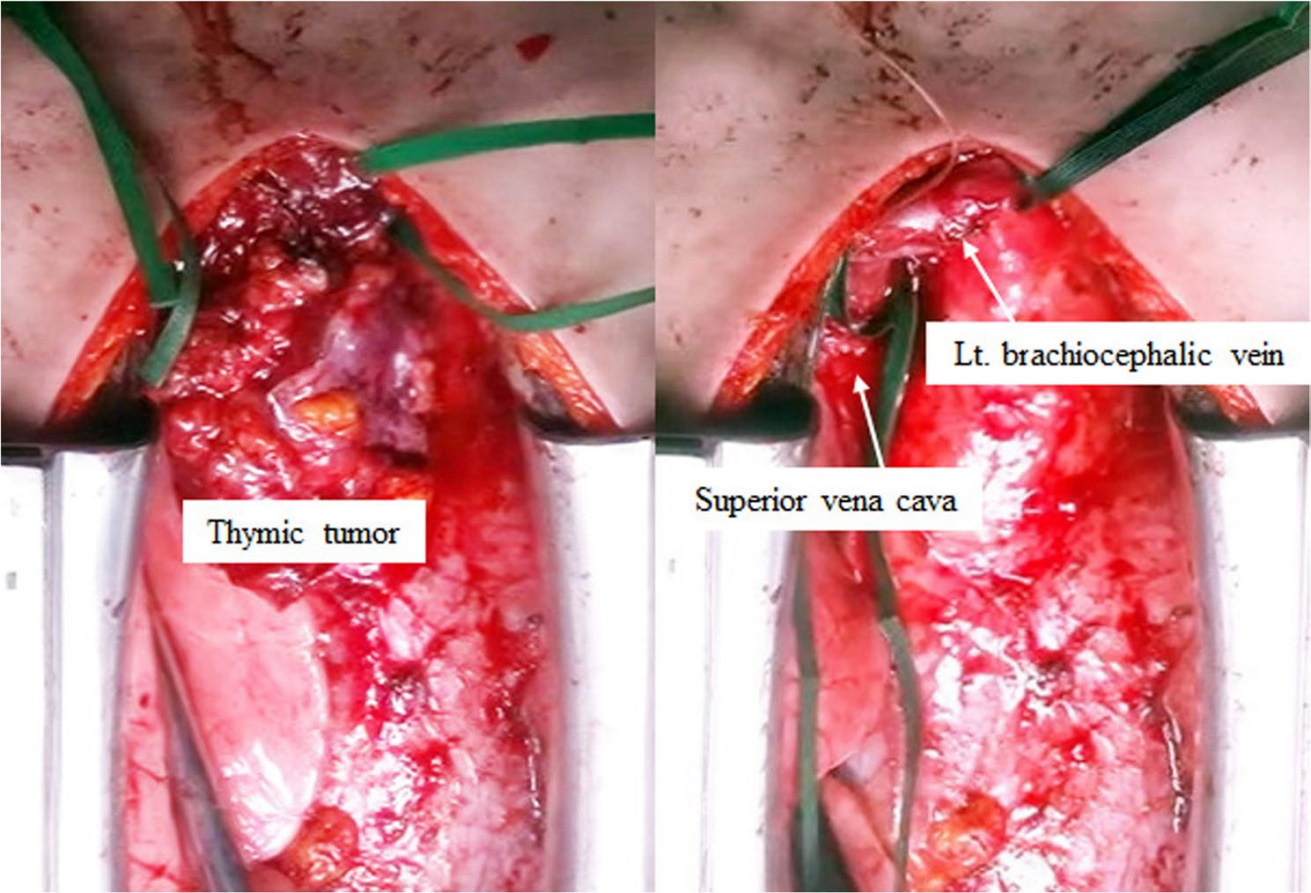
Surgical findings: A median sternotomy was performed for the thymectomy. Although firm adhesion to the left brachiocephalic vein was observed, sharp dissection was possible without the need for vascular reconstruction

Based on these findings, a decision was made to perform surgical intervention for both the diagnosis and the treatment of the patient’s condition. The anterior mediastinum was approached via a median sternotomy, with a thymectomy then performed. The tumor demonstrated notable adhesion properties, firmly adhering to the left brachiocephalic vein and pericardium, which significantly complicated the surgical procedure. After encircling and taping the left brachiocephalic vein and the superior vena cava, the procedure was subsequently performed under conditions that allowed for vascular reconstruction. However, by meticulously performing the surgical procedure with scissors, we were able to successfully complete the thymectomy without the necessity of vascular reconstruction or pericardial resection, and without leaving any residual tumor (Fig. 3). The sternum was closed using SternalPlate (Stryker, Michigan, USA), resulting in no sternal movement and minimal pain. The patient had no postoperative complications and progressed normally. The chest drain was removed on the third day after surgery, and the patient was deemed ready for discharge on the fourth day after surgery. However, she was not discharged until the seventh day after surgery due to family circumstances.

**Fig. 4:**
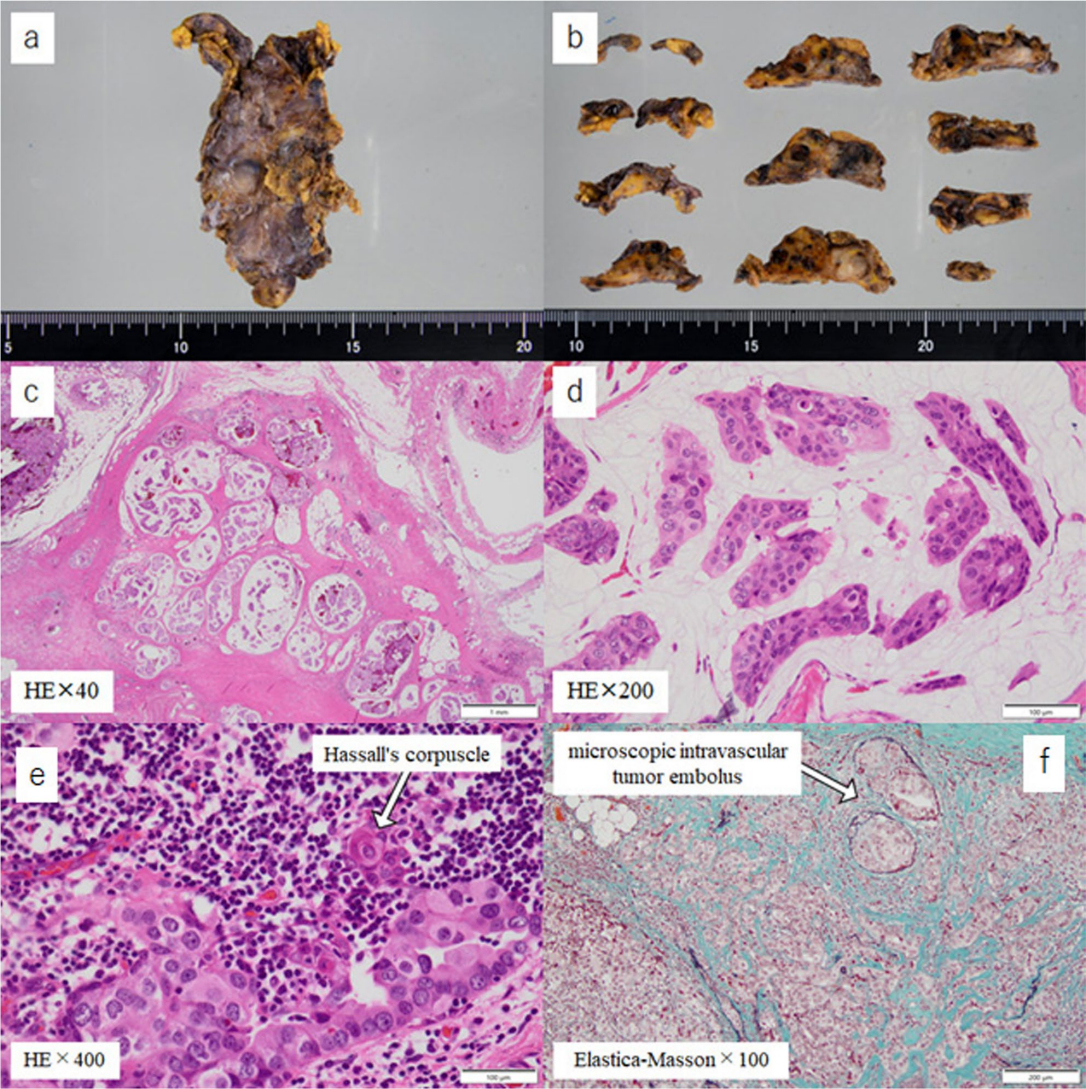
**a**, **b **Multiple solid nodules were observed in the thymus. **c**, **d **Atypical cells were invasively proliferating in a papillary pattern **e** Hassall’s corpuscles were identified in the vicinity of the tumor proliferation **f** A multitude of tumor clusters were identified within the blood vessels of the tumor

**Fig. 5:**
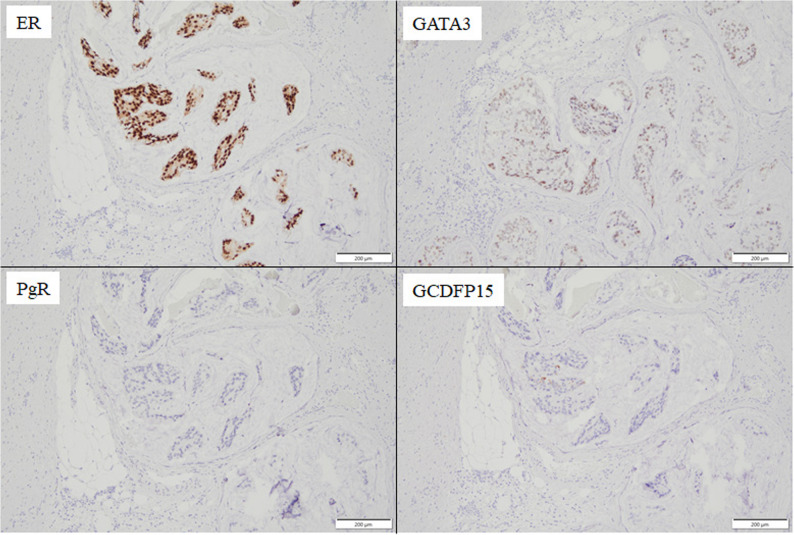
Immunohistochemistry showed positivity for ER and GATA3, and negativity for PGR and GCDFP15

Macroscopically, the excised specimen measured 48 × 27 × 20 mm, with the tumor itself measuring 40 × 25 × 13 mm, indicating that the tumor had almost completely occupied the thymus. A pathological diagnosis via hematoxylin and eosin (HE) staining revealed invasive proliferation of atypical cells in a papillary pattern within the thymus (Fig. 4). This finding suggested that the metastasis originated from a previous breast cancer. Furthermore, the presence of Hassall’s corpuscles in proximity to the tumor proliferation area confirmed that the metastatic organ was the thymus. Additionally, the presence of numerous tumor masses within the tumor’s blood vessels indicated that the mechanism of metastasis was hematogenous. Immunohistochemistry revealed positive results for ER and GATA-binding protein 3 (GATA3), and negative results for PGR and gross cystic disease fluid protein 15 (GCDFP-15) (Fig. 5). This led to a final diagnosis of isolated thymic metastasis of breast cancer. The results regarding biomarkers were as follows: ER Allred 5 + 3 = 8, PGR Allred 0, HER2 score 1+, and Ki-67 index 40%. Postoperatively, the patient is currently being treated with aromatase inhibitor (AI) and cyclin-dependent kinase 4/6 (CDK4/6) inhibitor. In the eight months since the patient underwent thymectomy, no local recurrence or distant organ metastasis has been detected, including on CT imaging.

## Discussion and conclusions

To date, no data has been published that directly compares the frequency of late isolated metastasis across various solid cancers. However, large-scale meta-analyses have demonstrated that for breast cancer, particularly ER-positive breast cancer, the risk of recurrence persists for 15 years (5 to 20 years post-treatment) even after the completion of 5 years of endocrine therapy [9].

The recurrence of breast cancer primarily occurs in distant organs, including bone, lung, liver, and brain, exhibiting distinct metastatic patterns depending on the immunohistochemical subtype. Luminal A (ER + and/or PR+, HER2–) tumors tend to relapse late and preferentially in the bone, while Luminal B (ER + and/or PR+, HER2+) and HER2-enriched subtypes (ER–, PR–, HER2+) more often metastasize to the liver and brain. Basal-like (triple-negative, ER–, PR–, HER2–) cancers relapse early, with a tendency toward lung and brain metastasis, underscoring the prognostic and clinical value of the molecular subtype classification [10].

Furthermore, late recurrence tends to involve single-organ metastasis. Ito et al. reported that 77.1% of patients with late recurrence more than seven years after surgery had single-organ metastasis, for which the most common type was found to be bone metastasis [11].

What, then, makes breast cancer particularly prone to late solitary metastasis? It is well established that breast cancer cells disseminate into the bloodstream and bone marrow at a relatively early stage of tumor development. Among these, ER-positive breast cancer cells exhibit a heightened propensity for long-term survival following the eradication of the primary tumor. These disseminated tumor cells (DTCs) have the capacity to evade immune surveillance and persist in a quiescent, non-proliferative state within specialized local microenvironments, or “niches,” such as the bone marrow. The protective effect of these niches may enable dormant tumor cells to remain viable for extended periods, potentially spanning years or even decades. Eventually, alterations in the local microenvironment can trigger their reactivation and proliferation. This reawakening of dormant DTCs frequently results in metastasis confined to a single organ, which is most commonly the bone, a clinical manifestation known as late solitary metastasis. This phenomenon is hypothesized to represent the reactivation of previously dormant cells rather than new dissemination events [12, 13].

Metastatic involvement of the thymus is exceedingly rare among solid malignancies, including breast cancer, and has been described almost exclusively in isolated case reports. Several mechanisms have been proposed to account for this rarity. First, the thymus constitutes a highly immunologically active microenvironment, serving as the site of T-cell maturation, which is thought to render it hostile to colonization by disseminated tumor cells [14]. Second, the blood–thymus barrier in the thymic cortex may act as a structural obstacle that prevents hematogenous tumor cells from extravasating and engrafting [15]. Third, unlike bone marrow or liver, the thymus is not considered to provide a permissive pre-metastatic niche capable of supporting long-term survival or reactivation of disseminated tumor cells [16]. Although these hypotheses are repeatedly cited in published case reports and small series, direct mechanistic evidence in humans remains limited.

Indeed, as described in the Background section, thymic metastasis from breast cancer is an exceedingly rare occurrence, even among intrathoracic metastatic sites. Furthermore, asynchronous solitary metastasis to the thymus—excluding cases that occur concurrently with the primary breast cancer or those accompanied by metastases to other organs—is even rarer. In cases of thymic tumors that develop at a later stage following breast cancer surgery, it is essential to differentiate them from other thymic tumors, such as thymoma, thymic carcinoma, malignant lymphoma, germ cell tumors, or thymic hyperplasia. Non-invasive diagnosis can effectively be achieved through the use of a percutaneous CT-guided needle biopsy. However, in the present case, the patient was a thin woman with a narrow mediastinum, which prevented the performance of a safe needle biopsy without penetrating the lung. Consequently, surgery was contemplated from the outset as both a diagnostic and therapeutic possibility.

As a result of the rarity of thymic metastasis in breast cancer, no standard treatment methods have been established. The resection of metastatic breast cancer to other organs is typically performed for two purposes: first, to distinguish metastatic tumors from primary tumors, and second, to identify biomarkers that can guide the selection of chemotherapy treatments. To date, no prospective randomized controlled trial has demonstrated a causal relationship between surgical resection of oligometastasis in breast cancer and improvement in prognosis [17]. However, a prospective, registry-based, multi-institutional observational study has been reported [18]. The findings of this study indicated that for patients with limited metastatic disease, particularly to the lung or liver, who were eligible for surgical resection or local intervention, the incorporation of surgery or local therapy into systemic treatment regimens led to a substantial reduction in the hazard ratio for mortality when compared with systemic therapy administered in isolation. In addition, this effect was most evident in younger patients (aged < 55 years), those with a prolonged metastasis detection-free interval (>24 months), and those with luminal A/B or HER2-positive subtypes. These findings suggest that, through judicious patient selection, surgical or local treatment for oligometastatic lesions may contribute to improved prognosis. Furthermore, in cases of thymic metastasis of breast cancer, long-term survival has been achieved in some cases through resection and subsequent appropriate chemotherapy [3, 7]. Our patient is currently undergoing treatment with AI and CDK4/6 inhibitor administration postoperatively, and there has been no recurrence as of eight months postoperatively. Nevertheless, meticulous subsequent observation is imperative to assess the progression of the disease.

## Data Availability

Not applicable.

## Abbreviations

ER: Estrogen receptor
PGR: Progesterone receptor
HER2: Human epidermal growth factor receptor 2
FEC: Fluorouracil + epirubicin + cyclophosphamide
TAC: Docetaxel + doxorubicin + cyclophosphamide
TAM: Tamoxifen
CEA: Carcinoembryonic antigen
FDG-PET: 18 F-fluorodeoxyglucose positron emission tomography
SUVmax: Maximum standardized uptake value
CT: Computed tomography
CA15-3: Cancer antigen 15 − 3
SCC-Ag: Squamous cell carcinoma antigen
CYFRA 21 − 1: Cytokeratin 19 fragment
NSE: Neuron-specific enolase
ProGRP: Pro-gastrin-releasing peptide
AFP: Alpha-fetoprotein
sIL-2R: Soluble interleukin-2 receptor
HE: Hematoxylin and eosin
GATA3: GATA binding protein 3
GCDFP15: Gross cystic disease fluid protein 15
AI: Aromatase inhibitor
CDK4/6: Cyclin-dependent kinase 4/6
